# Prebiotic potential of enzymatically prepared resistant starch in reshaping gut microbiota and their respond to body physiology

**DOI:** 10.1371/journal.pone.0267318

**Published:** 2022-05-16

**Authors:** Anum Khan, Huma Ali, Ubaid Ur Rehman, Ali Osman Belduz, Amna Bibi, Mujib Abdulkadir Abdurahman, Aamer Ali Shah, Malik Badshah, Fariha Hasan, Ali Osman Kilic, Asad Ullah, Sarwat Jahan, Muhammad Maqsood Ur Rehman, Rashid Mansoor, Samiullah Khan

**Affiliations:** 1 Department of Microbiology, Faculty of Biological Sciences, Quaid-i-Azam University, Islamabad, Pakistan; 2 Department of Biology, Faculty of Sciences, Karadeniz Technical University, Trabzon, Turkey; 3 Department of Medical Microbiology, Faculty of Medicine, Karadeniz Technical University, Trabzon, Turkey; 4 Department of Zoology, Faculty of Biological Sciences, Quaid-i-Azam University, Islamabad, Pakistan; 5 Department of Statistical Science, University College London, London, United Kingdom; Sejong University, REPUBLIC OF KOREA

## Abstract

The increase in consumer demand for high-quality food products has led to growth in the use of new technologies and ingredients. Resistant starch (RS) is a recently recognised source of fibre and has received much attention for its potential health benefits and functional properties. However, knowledge about the fate of RS in modulating complex intestinal communities, the microbial members involved in its degradation, enhancement of microbial metabolites, and its functional role in body physiology is still limited. For this purpose, the current study was designed to ratify the physiological and functional health benefits of enzymatically prepared resistant starch (EM-RSIII) from maize flour. To approve the beneficial health effects as prebiotic, EM-RSIII was supplemented in rat diets. After 21 days of the experiment, EM-RSIII fed rats showed a significant reduction in body weight gain, fecal pH, glycemic response, serum lipid profile, insulin level and reshaping gut microbiota, and enhancing short-chain fatty acid compared to control. The count of butyrate-producing and starch utilizing bacteria, such as *Lactobacillus*, *Enterococcus*, and *Pediococcus genus* in rat’s gut, elevated after the consumption of medium and high doses of EM-RSIII, while the *E*. *coli* completely suppressed in high EM-RSIII fed rats. Short-chain fatty acids precisely increased in feces of EM-RSIII feed rats. Correlation analysis demonstrated that the effect of butyrate on functional and physiological alteration on the body had been investigated during the current study. Conclusively, the present study demonstrated the unprecedented effect of utilising EM-RSIII as a diet on body physiology and redesigning gut microorganisms.

## Introduction

In western society fibre consumption has been significantly lowered and is far lower than the suggested level [1]. The key reason has been the change in lifestyle, which has promoted a significant reduction in vegetables, fruits and legume consumption [2]. With the goal of increasing fibre consumption in the diet, many fibre-enriched foods have been cultivated. One example of such fibre-rich food is enzymatically modified resistant starch (EM-RSIII) which has the potential to be ruled as preventing agent for health-related problems. Enzymatically modified resistant starch has pronounced prebiotic potential for promoting probiotic proliferation and having unique physicochemical characteristics to control satiety [3]. EM-RSIII has the ability to resist assimilation in the small intestine and reached the large intestine where it is fermented by bacteria, which results in the release of short-chain fatty acids (SCFAs), reduced fecal pH, and increased fecal volume. EM-RSIII is also associated with other additional imperative physiological health benefits, including reducing blood glucose and insulin level, plasma lipid and cholesterol level, and positively affecting colonocytes proliferation [4]. EM-RSIII has the functional characteristics to: resist hydrolysis by mammalian pancreatic α-amylases, resist the low pH of the stomach, the ability to be fermented by intestinal microbiota and selectively stimulate the growth and activity of intestinal bacteria associated with host health and overall well-being [5], resist absorption in the upper gastrointestinal tract. The significance of exhibiting direct health benefits because of bacterial fermentation and their metabolites is still the driving mechanism for all prebiotics and the focus of the current study.

As previously reported, a diet rich in rapidly digestible starch results in an elevation of insulinemic and glycemic responses, associated with poor satiety and enhanced energy intake [6, 7]. Therefore, changes in the quality and type of carbohydrates consumed have a great impact on human health. Therefore, incorporating EM-RSIII in diet preparations is an excellent approach to reducing the glycemic index of food [8]. Moreover, a negative correlation has been found between high-density lipoprotein cholesterol and dietary glycemic index. Therefore, the low glycemic index of food having a significant consideration, intended to upgrade the metabolic control of hyperlipidemia, specifically in the diet formulation for people with diabetes [9]. Prebiotic-rich foods may be the best method for overcoming and preventing the adverse effects of foods with high glycemic reactions [10]. Most essential microbial metabolites such as short-chain fatty acids (SCFAs) are primarily produced through microbial fermentation from food products in the gut. Short-chain fatty acids include acetate, propionate, and butyrate, which are generated by specified genera of microorganisms. The significant contribution of SCFAs is to control the host’s appetite for bodyweight management. Propionate production stimulates gut hormones by activating its receptors (FFAR2, Free fatty acid receptor 2; FFAR3, Free fatty acid receptor 3), which reduces appetite. Propionate along with butyrate regulate appetite-regulation hormone through neuropeptide Y (NPY) expression in hypothalamus through stimulation of glucagon-like peptide 1 (GLP-1) and peptide YY (PYY) hormones in the colon. Butyrate is also involved in regulation of energy metabolism through enhancing lipolysis and the suppression of leptin as appetite suppressing hormone from adipose tissues. Therefore, SCFAs have a pivotal mediating and regulating role leading to the beneficial effect of EM-RSIII. The traditional prebiotics, including fructooligosaccharides, inulin, and galactooligosaccharide, still provide evidence of beneficial health effects [11] due to their fermentation and assessable literature. However, many other categories of food ingredients that may equal or even more effective than the traditional prebiotics need to be explored for their physiological and functional benefits.

Therefore, the current study was undertaken to introduce a new food ingredient as a potential prebiotic prepared through enzymatic hydrolysis [12] as a critical player to explore the interlink between prebiotic intake, defining the consequences of EM-RSIII on the gut microbiome re-structure and SCFAs production and its effect on blood profile and body physiology. The goal of the current work was to study the effect of different percentages of newly enzymatically prepared resistant starch (EM-RSIII) present in the standard control diet on the rat’s body weight, fasting and postprandial blood glucose level, fecal pH, its effects on serum lipid profile, serum insulin level, modulation in gut microbiota, their metabolites and cecum health of control and EM-RSIII fed rats to declare it as safe prebiotic. Additionally, the correlation analysis of these effects was also made to examine the mediating roles of SCFAs and gut microbiota in EM-RSIII fed rats.

## Materials and methods

### Rats and feeding regiments

Twenty Sprague Dawley female rats of two months age and standard diet were provided by the Department of Zoology, primate facility Quaid-i-Azam University Islamabad, Pakistan. Rats were randomly divided into four groups in separate cages in a specific pathogen-free environment and placed in an air-conditioned room provided with food and water for 21 days [13]. Rats were sacrificed by cervical trauma and all efforts were made to minimise their suffering. The guideline given by the National Institute of Health regarding the treatment and care protocol were followed while the guide for the use and care of laboratory animals was approved by the Institutional Animal care and use committee at the Quaid-i-Azam University Islamabad.

### Experimental design for EM-RSIII feeding

Randomly the rats were allocated into four groups: the control group did not receive EM-RSIII in a standard diet, and the other groups were given low-EM-RSIII (2 g/kg body weight), medium-EM-RSIII (4 g/kg body weight), and high-EM-RSIII (8 g/kg body weight), mixed in the standard control diet [13] and each group-housed in wire cages in a climate-controlled environment with free access to feed and water. The temperature was maintained at 21°C ±1°C with alternating 12-hour periods of light and darkness.

### Chemicals

The chemicals used were 30% Hydrogen peroxide (Suprapur^®^Merck USA), N,N,N′,N′-tetramethyl-p-phenylenediamine dihydrochloride (Thermo Scientific™, USA), Agarose (Bio-Helix China), Man rogosa sharp agar (GranuCult^®^, USA), Rogosa agar (Thermo Scientific™), 1492R, 5’-GGTTACCTTGTTACGACTT-3’ and 27F, 5’-AGAGTTTGGATCMTGGTCAG-3’ (Macrogen, South Korea), PCR master mix (5x HOT FIREPol^®^ Blend Master Mix), 100 bp DNA marker (Solis BioDyne, Estonia), and Big Dye ^®^ (Applied Biosystems, USA).

### Analytical methods for measuring the effect of EM-RSIII fed on rats physiology

#### Body weight measurement

Each rat’s body weight was measured every third day of the experiment by using a weighing balance.

#### Measurement of fecal pH of rats

The fecal pH of rats from each group was recorded by preparing a suspension of one gram of freshly collected feces in 10 mL of distilled water using a digital pH meter.

#### Measurement of the blood glucose level of rats

At the start of the experiment the blood samples were taken from each rats’ tail vein at fasting, 40 min, and 120 min after consuming the feed to measure serum glucose level and then on the last three days of the experiment at fasting, 40 min, and 120 min after consuming the feed using a glucometer.

#### Measurement of cecum length and weight of rats

Cecum from all groups was excised after dissection, their length and weight were measured. Cecum content was collected in a separate tube and stored at -20°C for further analysis.

#### Measurement of the serum lipid profile of rats

Before rat’s dissection, 2 mL of blood from the heart of each group of rats were collected by cardiac puncture, in syringe in separate gel tubes and immediately centrifuged for 5 min at 800 rpm. Serum was collected in separate serum cups, which were further processed for lipid profile. Serum cholesterol and triglycerides were measured using 10 μL serum samples with 1 mL cholesterol reagent and 1 mL triglyceride reagent from the kit (cholesterol and triglycerides liquid Color kit) separately incubated for 5 min at 20–25°C, and absorbance values were spectrophotometrically recorded at 546 nm (using “microlab 300” an analytical instrument). HDL and LDL levels were determined according to the following formulas:

**HDL determination:** HDL = Cholesterol/5

**LDL determination:** LDL = Triglyceride/5+HDL-Cholesterol

#### Measurement of serum insulin level of rats

Before rat’s dissection, blood samples were collected from rats of each group separately in gel tubes as in the case of serum lipid profile and immediately centrifuged at 800 rpm for 5 min. Serum was collected in serum cups and analysed for insulin level using an automated chemistry analyzer (Architect C4000, Abbott) [14].

#### Histopathology of rat’s colon

After 21 days of the experiment, rats were killed by cervical dislocation then the blood sample was taken by cardiac puncture for blood analysis and then dissecting all the rats one by one, the colon of each group of rats was removed, cleaned with normal saline, and stored in 70% formalin. Segments were fixed in 10% formalin for 48 hours, dehydrated with ethanol, and embedded in paraplast medium. Cross-sections with a thickness of 7 μm were stained with hematoxylin and eosin (Thermo, Shandon finesse 325, UK). Histological sections were examined under the conventional microscope (Olympus, Shinjuku-ku, Tokyo, Japan) at 40X resolution.

### Microbial analysis from rat’s cecum and fecal samples

Microbiological analysis was performed from luminal contents; weighed, homogenised preserved cecal and fecal samples were serially diluted in sterile peptone water. Homogenates of 10-fold serial dilution were plated on specific media De Man, Rogosa and Sharpe agar (MRSA) and Rogosa agar, modified Columbia and Modified Yeast extract, Casitone and Fatty acid (YCFA) media used for the enumeration of probiotic bacteria. The samples were also checked for *E*. *coli* by spreading on eosin methylene blue agar (EMB). All the media plates were anaerobically incubated in a sealed jar for 48 hours at 37°C.The colonies on the various selective media were noted and counted after incubation and chosen colonies were further purified for identification. The finishing count of colonies were reported as colony forming units (CFU) from each sample. Probiotic strains were isolated and identified based on colony morphology, biochemical analysis, and 16S rRNA sequencing.

### Quantification of SCFAs levels in fecal samples of experimental rats

The investigation of SCFAs in fecal samples was performed by gas chromatography (Shimadzu Corporation, GC-2014C, Japan) equipped with a DB-FFAP capillary column (30 m × 0.25 μm × 0.25 mm) (Agilent Technologies, Wilmington, DE, USA) and flame ionisation detector. The standard of SCFAs (acetate, A116173; propionate, P110445; butyrate, B11se0438; isobutyrate, I103524; valerate, V108269; isovalerate, I108280;) purchased from Aladdin Bio-Chem Technology Co., LTD (Shanghai, China) were used for quantification. For SCFA measurement, 200 mg faecal sample was homogenised with 1 mL distilled water; then 0.15 mL dichloromethane (w/w) and 1.6 mL ethyl acetate were added; samples were wobbled at 4°C for 10 min and centrifuged at 10,000 rpm for 10 min; the organic phase was collected, filtered (0.22 μm, NYLON6) and analysed by gas chromography. The carrier gas was hydrogen at a 45 mL/min rate, and the samples were injected in split mode with a volume of 1 μL. After injecting each sample, the injection was appropriately washed with methanol. The initial oven temperature was 120°C, and end temperature was 200°C, rate of change was 10°C/min, the total run time was 10 min, the back inlet was set at 200°C, 25.5 psi pressure, and 24.4 mL/min flow rate. GC was equipped with a Flame ionisation detector with an ’ON’ thermal zone, temperature 300°C, makeup flow 15 mL/min, H_2_ flow 45 ml/min, and 149.9 ml/min airflow.

### Statistical analysis

Following a completely random design the experiment was performed, in which the animals were the experimental units. Differences among experimental groups were evaluated by Duncan’s multiple range test (SAS Institute, Cary, NC, USA) following ANOVA. The correlations among the butyrate in feces levels and other biochemical indexes, including body weight, blood glucose, serum insulin, triglycerides, and cholesterol level, and relative abundance of intestinal microorganism, were evaluated by Spearman correlation analysis using R software. All data are presented in the text as mean ± SEM. Different lowercase letters (a, b, c) indicate the significant differences among different groups considered significant at p < 0·05.

## Results

This work is the continuation of our previous research work where resistant starch type III was prepared enzymatically and named EM-RSIII [12]. The diet was prepared by supplementing the EM-RSIII to standard control diet in a specific ratio to prepare a diet with zero (control), low, medium, and high EM-RSIII in order to exploit the prebiotic nature of EM-RSIII. This diet was fed to rats for 21 days. The effects of the EM-RSIII diet on rat’s physiology, blood glucose level, fecal pH, serum insulin, cholesterol level, and colon microbiota of rats were observed during the current study.

### Body weight of rats

There was no toxic or diarrheal effect on rats after the administration of EM-RSIII supplemented diet and control diet. Though, all EM-RSIII fed rats exhibited a reduced weight gain compared to the control group, which was evident after 2 weeks of receiving the EM-RSIII diet. This result was most possibly related to lower food intake observed in all low, medium and high EM-RSIII fed rats in comparison with the control group. Throughout the experimental period the rats were in good health. The average body weight of all the rats was 170 g initially, and after 21 days of experiment average weight gain of high EM-RSIII fed rats (208 g) was significantly lower than that of control (250 g). The same trend was followed in the case of low and medium EM-RSIII rats compared to control. The average weight gains of rats were in the order of control > low-fed EM-RSIII > medium-fed EM-RSIII > high-fed EM-RSIII as demonstrated in Table 1.

**Table 1:** Average body weight gain of rats at the start and end of the experiment.

Groups	Body weight(g) initial	Body weight(g) final
**Control**	170 ± 2 a	250 ± 2 a
**Low RS III**	170 ± 1 a	243 ± 4 b
**Medium RS III**	170 ± 3 a	230 ± 3 c
**High RS III**	170 ± 1 a	208 ± 4 d

Mean (n = 5) with the same letter in a column within same water regime are statistically similar at p< 0.05 according to Duncan’s multiple range test. Low RS = 2g/100g, Medium RS = 4g/100g, High RS = 8g/100g.

### Blood glucose level of rats

The blood glucose level examined throughout the experiment indicated the diet-specific response towards starch molecules supplemented in food formulations. The blood glucose level of high EM-RSIII fed rats was significantly decreased when measured at the end of the experiment compared to control S1 Table. The average blood glucose level values can be articulated as control ˃ low EM-RSIII fed rats ˃ medium EM-RSIII fed rats ˃ high EM-RSIII fed rats. At the start of the experiment, blood glucose levels for all the groups were analysed at fasting, 40 min, and 120 min after consuming EM-RSIII containing feed. Blood glucose level was observed for all rats, i.e., on fasting have 117, 113, 106, and 104 mg/dL for control, low, medium, and high EM-RSIII fed rats respectively. After 40 min of consuming feed, the blood glucose level becomes 125, 121,113, and 111 mg/dL, respectively, and then after 120 minutes, the blood glucose level become 132, 125, 118, and 116mg/dL for control, low, medium and high EM-RSIII respectively indicated that at the start of the experiment the blood glucose level was high even from the normal blood glucose level, i.e. (70-110mg/dL) of all experimental rats [15]. But after EM-RSIII feed, the increase in blood glucose level of rats seems to be inversely proportional to the amount of EM-RSIII in rat’s diet with alternate time periods as shown in Table 2. Results shows that there was a noteworthy reduction in the blood glucose level of EM-RSIII fed rats, especially the rats with a high amount of EM-RSIII feed attributed to the diet-specific response of blood glucose due to differences in the type of starch molecules, arrangement, and specific structure.

**Table 2:** Average blood glucose level mg/dL of rats at different time intervals, i.e., fasting, 40 min, and 120 min at the start and end of the experiment.

Time (min)	Groups	Initial blood glucose level mg/dL	Final blood glucose level mg/dL
**0**	**Control**	113.8 ± 0.37 a	116 ± 0.05 a
	**Low RS**	123.6 ± 0.68 b	113 ± 0.09 b
	**Medium RS**	131.8 ± 0.37 c	97 ± 0.019 c
	**High RS**	113.4 ± 0.40 a	91 ± 0.06 d
**40**	**Control**	123.8 ± 0.58 b	125 ± 0.13 a
	**Low RS**	131.4 ± 0.24 c	118 ± 0.69 b
	**Medium RS**	113.6 ± 0.24 a	110 ± 0.03 c
	**High RS**	123.8 ± 0.37 b	104 ± 0.11 d
**120**	**Control**	131.2 ± 0.20 c	134 ± 0.14 a
	**Low RS**	113.6 ± 0.24 a	123 ± 0.18 b
	**Medium RS**	123.4 ± 0.24 b	116 ± 0.05 c
	**High RS**	131.4 ± 0.24 c	108 ± 0.07 d

Mean (n = 5) with the same letter in a column within same water regime are statistically similar at p< 0.05 according to Duncan’s multiple range test. Low RS = 2 g/100g, Medium RS = 4 g/100g, High RS = 8 g/100g.

### Fecal pH of rats

Administration of EM-RSIII containing diet affected the pH values of intestinal contents of rats significantly. Reduction in pH of cecum and colon contents was observed as compared to control-fed rats. The average fecal pH at the first three days of the experiment was 8.0, but after 21 days, the pH of high EM-RSIII fed rats was significantly dropped to 6.5 compared to the initial pH of experimental days, as seen in Table 3. A similar fashion of decreased pH in fecal samples related to experimental diet was observed in low and medium EM-RSIII fed rats samples while in the case of control pH remains the same (pH 8) from the start till the final days of the experiment. The pyramid of pH values can be expressed as control ˃ low EM-RSIII fed ˃ medium EM-RSIII fed ˃ high EM-RSIII fed rats.

**Table 3:** Average fecal pH at start and end of the experiment.

Groups	Fecal pH (initial)	Fecal pH (final)
**Control**	8 ± 0.1 a	8.075 ± 0.015 a
**Low RS**	8 ± 0.2 a	7.58 ± 0.015 b
**Medium RS**	8 ± 0.3 a	6.8 ± 0.1 c
**High RS**	8 ± 0.4 a	6.5 ± 0.15 d

Mean (n = 5) with the same letter in a column within same water regime are statistically similar at p< 0.05 according to Duncan’s multiple range test. Low RS = 2g/100g, Medium RS = 4g/100g, High RS = 8g/100g.

### Cecum length and weight of rats

The average weight and length of the cecum was measured after the rat’s dissection. Significant increase in weight and length of EM-RSIII fed rats were observed with the high EM-RSIII fed group showing the highest cecum length (6.35cm) and weight (1.42 g) as compared to cecum length (5.8cm) and cecum weight (1.25 g) of control group rats. Low and medium EM-RSIII fed rats cecum sample also showed the healthy, lengthier, and weighed cecum compared to control fed rats as shown in Table 4. The average cecum length and weight were in the order of control < low EM-RSIII fed rats < medium EM-RSIII fed rats < high EM-RSIII fed rats.

**Table 4:** Average cecum length and weight after dissecting the rats and average serum cholesterol, triglyceride, LDL, HDL, and insulin level from rats’ blood samples.

Parameters studied	Control	Low RS	Medium RS	High RS
**Cecum length (cm)**	5.8 ± 0.1 b	6 ± 0.1 b	6.305 ± 0.005 a	6.35 ± 0.15 a
**Cecum weight (g)**	1.25 ± 0.05 c	1.235 ± 0.005 d	1.39 ± 0.01 b	1.42 ± 0.01 a
**Serum cholesterol level (mg/dL)**	87 ± 1 a	84 ± 1 b	80 ± 1 c	77 ± 1 d
**Serum triglycerides level (mg/dL)**	288 ± 2 a	154 ± 2 b	119 ± 1 c	107 ± 1 d
**Serum LDL level (mg/dL)**	45 ± 1 a	43 ± 2 a	39 ± 1 c	33 ± 1 d
**Serum HDL level (mg/dL)**	16 ± 2 b	16.5 ± 0.5 b	17 ± 1 b	22 ± 2 a
**Serum insulin level (mU/mL)**	0.8 ± 0.1 a	0.4 ± 0.1 b	0.36 ± 0.14 b	0.345 ± 0.145 b

Mean (n = 5) with the same letter in a column within same water regime are statistically similar at p< 0.05 according to Duncan’s multiple range test. Low RS = 2 g/100 g, Medium RS = 4/100 g, High RS = 8 g/100 g.

### Serum lipid profile of rats

After cardiac puncture, rats blood samples were analysed, and results indicated a considerable decrease in serum triglyceride, cholesterol, and LDL values in the low and medium EM-RSIII fed groups, with a significant decrease observed in high EM-RSIII fed rats (33 mg/dL) as compared to control (45 mg/dL). On the contrary, an increase in serum HDL values was recorded in EM-RSIII fed rats as compared to control (16 mg/dL) with significantly increased HDL values in high EM-RSIII fed rats (22 mg/dL) as mentioned in Table 4. The average cholesterol, triglyceride, and LDL values changed positively in the following order: control ˃ low EM-RSIII fed rats ˃ medium EM-RSIII fed rats ˃ high EM-RSIII fed rats respectively while the average HDL values were control < low EM-RSIII fed rats < medium EM-RSIII fed rats < high EM-RSIII fed rats.

### Serum insulin level of rats

Serum insulin level was determined in blood samples, which revealed a significant decrease in the serum insulin levels of rats after supplementation with the maximum decrease observed for high EM-RSIII fed rats compared to control. The average insulin level of control was 0.8, while that of high EM-RSIII fed rats was 0.344 mU/mL. The decreasing trend was also observed in the case of low and medium EM-RSIII fed rats samples compared to control rats samples demonstrating the diet-specific response of insulin sensitivity. The drift of average insulin level was in the order of; control ˃ low EM-RSIII fed rats’ ˃ medium EM-RSIII fed rats’ ˃ high EM-RSIII fed rats as demonstrated in Table 4.

### Colon tissue histopathology of rats

**Fig 1:**
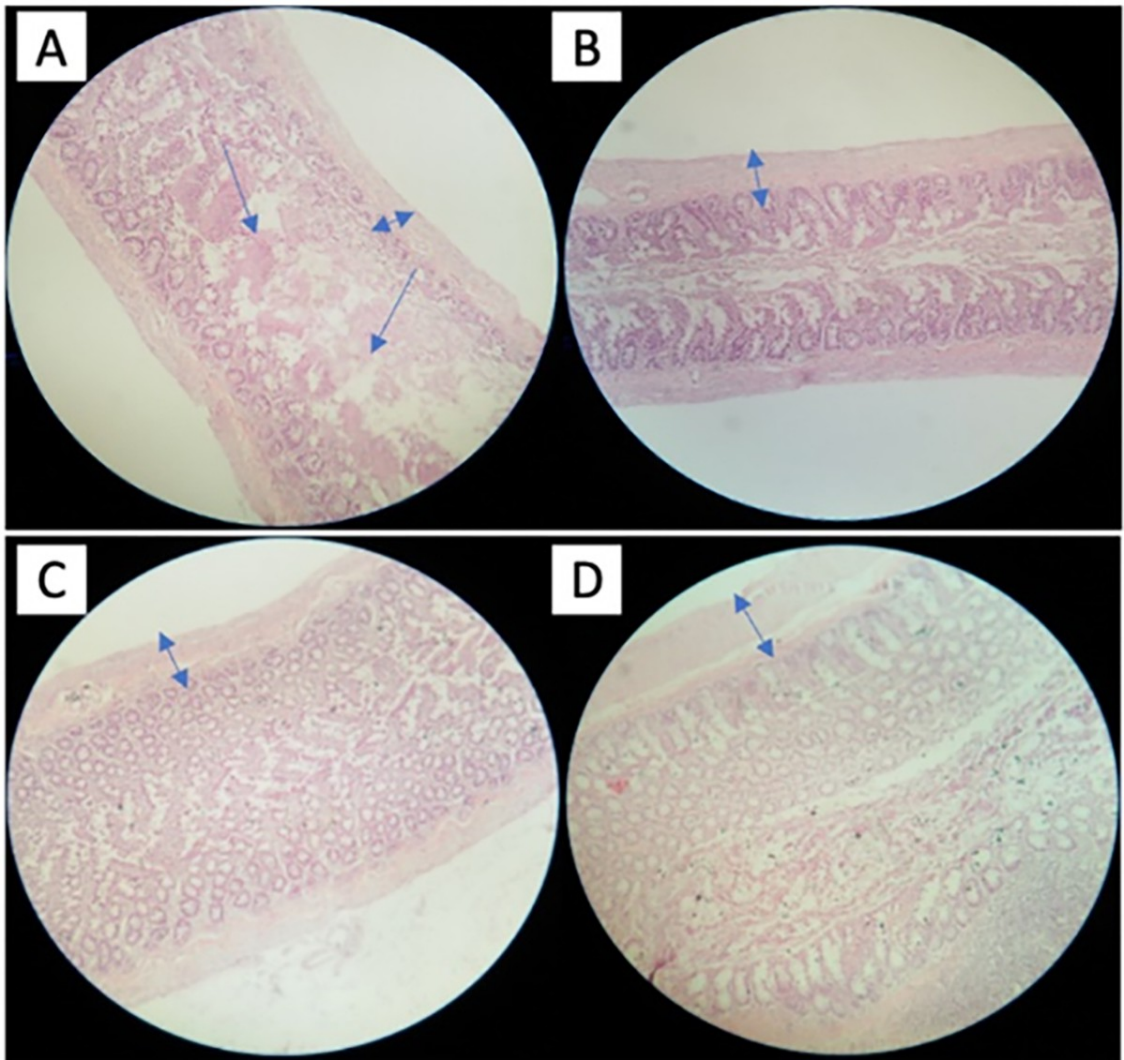
Colon histology of cecal rat’s samples. A: Colon histology of control group, B: Colon histology of low EM-RSIII fed group, C: Colon histology of medium EM-RSIII group, D: Colon histology of high EM-RSIII group.

The colonic tissue of the control group showed ulcer symptoms, and the mild ulcer was also seen in the colon tissue of low and medium EM-RSIII fed rats. Independent of the localisation and the overall extent of an inflammation, three main categories sufficiently reflected the severity of histopathology: (i) quality, and the dimension of inflammatory cell infiltrates, (ii) epithelial changes and (iii) overall mucosal architecture. Differences in mucosal linings were also observed in rats groups; the thin mucosal lining was observed in control in contrast to the healthy thick and intact mucosa of high EM-RSIII fed rats with prominent lymphocytic aggregates as shown in Fig 1. The damage order of the tissue was control˃ low EM-RSIII fed rats ˃ medium EM-RSIII fed rats ˃ high EM-RSIII fed rats.

### Microbial analysis of fecal and cecum content of rats

**Fig 2:**
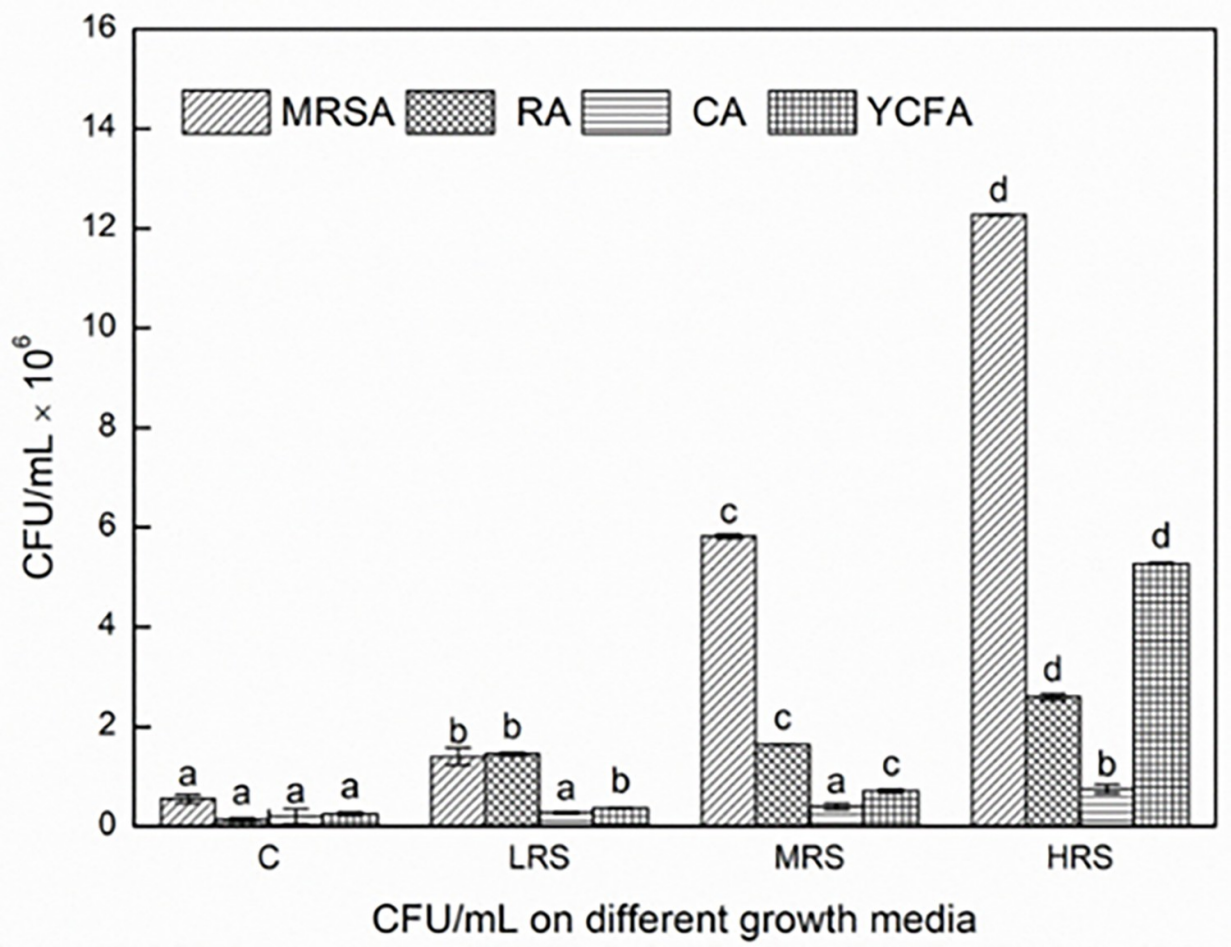
CFU/mL counted on the probiotic media used. (MRSA; De Man, Rogosa and Sharpe agar, RA; Rogosa agar, CA; modified Columbia agar and YCFA media used for the enumeration of probiotic bacteria.).

The microbiological analysis of fecal and cecum rat samples revealed that administering the EM-RSIII diet leads to modification of intestinal and fecal microbiota of rats as compared to the control group. The fecal and cecum contents analysis showed the high count of *lactobacilli* in high EM-RSIII fed rat samples compared to low EM-RSIII, medium EM-RSIII and control fed rat samples. Morphologically different types of colonies appeared on all specific media De Man, Rogosa and Sharpe agar (MRSA) and Rogosa agar, modified Columbia and Modified Yeast extract, Casitone and Fatty acid (YCFA) containing agar plates including white creamy smooth, opaque or transparent and some are small and large colonies also with smooth or irregular margins S1–S4 Figs. Biochemical analysis revealed that all the strains were gram-positive, anaerobes, oxidase, and catalase-negative. Twenty-four strains were sequenced from the rat’s fecal and cecum samples of control and EM-RSIII fed rats. Most of them were *Lactobacillus*, *Enterococcus*, and *Pediococcus* genus. Different *Lactobacilli* strains found were *Lactobacillus reuteri*, *Lactobacillus planterum*, *Lactobacillus gasseri*, *Lactobacillus johnsoni*, *Lactobacillus curvatus*. *Enterococcus faecium* and *Pediococcus pentosaceus* were also identified as shown in “Fig 2”. Most of the *lactobacillus* species were identified from high EM-RSIII fed rats samples, while in control samples, only *Enterococcus faecium* and *Lactobacillus curvatus* were detected. The rat’s samples were also checked for *E*. *coli* by spreading on eosin methylene blue agar (EMB) S5 Fig, the CFU/g of *E*. *coli* was higher in control as compared to low, medium, and high EM-RSIII fed rat’s samples. *E*. *coli* was identified by their metallic sheen color on EMB media. The CFU/g calculated was in the order of control ˃ low EM-RSIII fed rat sample ˃ medium EM-RSIII fed rat sample ˃ high EM-RSIII fed rat sample pattern as shown in Table 5.

**Table 5:** Different culture media used for quantitative isolation of microorganisms identified based on 16 sRNA.

Culture media	Main organisms counted
**Anaerobic culture**	
**MRS agar**	*Lactobacillus reuteri*, *Lactobacillus gasseri*, *Lactobacillus johnsoni*, *Pediococcus pentosaceus*, *Pediococcus acidilactici*, *Enterococcus faecium*, *Enterococcus durans*
**Rogosa agar**	*Lactobacillus plantarum*, *Lactobacillus curvatus*, and *Lactobacillus reuteri*
**Modified Columbia agar**	*Lactobacillus fermentum*, *Pediococcus acidilactici*
**Modified YCFA**	*Enterococcus faecium*, *Pediococcus pentosaceus*
**Aerobic culture**	
**Eosin methylene blue agar**	*E*. *coli*

### EM-RSIII feed improved the generation of SCFAs

The major microbial metabolites i.e. SCFAs generated by gut microbiota, were analysed in fecal samples by gas chromatography S6–S9 Figs. EM-RSIII feed significantly improved the butyrate propionate and acetate levels in the feces of the experimental group compared to the control rat group. Moreover, it was also observed that the great amount of butyrate-producing microbes, including *Lactobacillus*, *Enterococcus*, and *Pediococcus genus*, was as considerably enhanced in high EM-RSIII feed rats. These results revealed that EM-RSIII feed improved butyrate production in EM-RSIII fed experimental rats.

### Correlation analysis between the fecal/serum butyrate levels and other biochemical indexes

**Fig 3:**
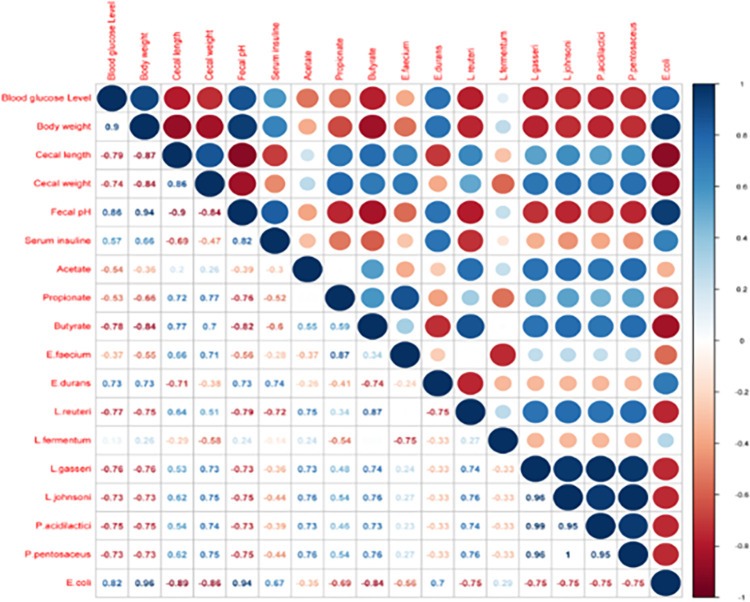
Correlation analyses between the fecal/serum butyrate levels and other biochemical indexes. In the upper-right part of the matrix, the size and color of the circles demonstrate the level of the correlation index (red representing a negative correlation, and blue representing a positive correlation) and the corresponding numeric value of the correlation index can be found in the right side.

Spearman’s correlation assessment was done based on these parameters to explain the correlation among the microbial metabolites, microbiota and other biochemical indexes. The correlation is indicated between the butyrate levels, and other biochemical indexes are given in Fig 3. For instance, the fecal butyrate levels, enhanced by high RS feed, exhibited a significantly negative correlation with bodyweight gain (r = -0.84), blood glucose level (r = -0.78), serum insulin (r = -0.62), fecal pH (r =, -0.82), and positively correlated with cecum length (r = 0.77) and cecum weight (r = 0.7).

## Discussion

Dietary fiber is not a "nutrient"; it is, on the other hand, a key player in our diet to modulate human health. Dietary fiber directly relates to "diabesity", which illustrates the mutually dependent relationship between diabetes and obesity. Roughly 80% of diabetic patients are identified with obesity. Dietary interventions can prevent diabesity, especially by incorporating an adequate amount of dietary fiber, including resistant starch (RS III) [16]. RS III has inspired researchers in the past few decades because of its numerous health benefits [12]. RS III persists undigested in the small intestine, and it is subjected to fermentation in the large intestine as compare to digestible starch. The current study expects to sum up the recent information related to the dietary RS III to prevent obesity and related outcomes. This study provides valuable findings relevant to directly assessing enzymatically modified resistant starch (EM-RSIII) from maize flour as a prebiotic on manipulation of gut microbiota and metabolic and physiological behavior of rats. The data disclosed that EM-RSIII induced substrate-specific shifts in the microbial population of fecal and colon samples that were closely associated with the utilization of food between the experimental and control group. The low weight gain in the rats fed with resistant starch, particularly in the high EM-RSIII fed rats, was observed compared to control, medium, and low EM-RSIII fed rats. There were many attributes of resistant starch as compared to normal starch which could promote body weight loss/weight maintenance includes reduce postprandial insulinemia and increase the level of gut satiety peptides, lesser fat storage in adipocytes, increase fat oxidation, and protection of lean body mass, and most important period of satiety [17]. Similar result was also reported by Brites CM *et al*, by the incorporation of RS as a feed for significantly reduced weight gain [13]. In addition to this, Shen *et al*, reported the study using Sprague–Dawley model concluded that RS could reduce body fat of high-fat diet-induced overweight and obese rats, and improve lipid metabolism disorders [18]. Conclusively with addition of scientific evidence, fermentable carbohydrate such as RS III as diet supplementation has been proved to boost up the release of gut hormones which functions in appetite regulation and possibly, leptin release for suppressing hunger increasing period of satiety [19, 20] and ultimately controlling body weight gain in high EM-RSIII-fed rats.

As the current study revealed, lower serum insulin levels and blood glucose levels accredited to the EM-RSIII fed were especially observed in rats fed with a high EM-RSIII diet compared to the control group. This decrease in glucose and insulin level is mainly attributed to glucagon-like peptide-1 (GLP-1) release in the gut. GLP-1 excreted by intestinal endocrine cells is mainly responsible for maintaining glucose homeostasis by improving insulin secretion, lowering plasma glucose concentration, and maintaining beta cell functions of the pancreas. The secretion of GLP-1 is associated with stimulation by a short-chain fatty acid, especially acetate and butyrate, which are the main product of resistant starch digestion by colonic bacteria is majorly the main reason for reduced glucose and insulin level in high EM-RSIII fed rats in the current study [21, 22]. Similar results were reported by Freeland and Wolever, that the diet rich in SCFAs is always needed to the hyper insulinemic subjects to increase concentration of GLP-1. The ability of SCFAs to induce GLP-1 secretion has been widely reported [23]. Furthermore, Coppola *et al*, evident that SCFAs provoke the secretion of GLP-1 in the plasma but the mechanism which links the production of SCFAs with the GLP-1 hormone is not fully understood [24]. In addition to this, Romaní-Pérez *et al*, recently presented that the primary colonic cultures expressing mRNAs for both FFAR2 and FFAR3 and the propionate and acetate also stimulate the secretion of GLP-1 by activating receptors (FFAR2) increase of intracellular calcium level [25]. Moreover according to Wang et al, it has been found that mice with deficient FFAR2 have significantly reduce GLP-1 protein content which shows that may be FFAR2 is involved in function of L-cells [26].

Lowering of glycemic response findings by EM-RSIII is in agreement with previous studies [27]. A study conducted by Aziz *et al*. concluded that a low glycemic response was produced in obese rats after the ingestion of high amylose-resistant corn starch, primarily because of starch resistivity [28]. Though, the same study found improved levels of the hormone incretin (which has antihyperglycemic properties) in high amylose resistant corn starch fed obese rats, both freely and in energy-restricted experimental groups. A number of human studies had reveal that the blood glucose response is reduced when native starch is substituted with resistant starch. Whereas, in the current study during correlation analysis, the negative correlation was observed in blood glucose (r = -0.78) and serum insulin level (r = -0.62) with that of butyrate representing the role of SCFA in maintaining blood glucose and serum insulin level. Compared with control, low, medium, and high EM-RSIII fed rats reduced the serum triglyceride and cholesterol level after the experimental rat’s blood samples analysis. Similar observations have been reported by the trials on feeding RS containing food like Adzuki bean starch (AS) and Tebou bean starch (TS) to rats showed that the high RS diet has more effect on lowering serum cholesterol due to increase the levels of hepatic SR-B1 (scavenger receptor class B1) and cholesterol 7alpha hydroxylase mRNA [29]. SR-B1 is a cell surface HDL receptor that mediates HDL-cholesteryl ester uptake in the liver, providing cholesterol for bile acid synthesis. Cholesterol 7 alpha-hydroxylase is essential for the oxidation and regulation of cholesterol levels in serum. Another study was conducted on hamsters fed with cassava starch extruded with 9.9% oat fiber and cassava starch extruded with 9.7% RS; results showed that food containing RS lowered serum cholesterol level and used as preventing agent for cardiovascular disease [30, 31].

In order to get better insight for understanding the effect of EM-RSIII on rat’s gastrointestinal health, histopathological examination using colon tissue samples was conducted. The description of mucosal pattern and histological observations showed some low-level infiltration of the colon in control, low and medium EM-RSIII fed rat compared to high EM-RSIII fed rat’s samples. A possible reason for the inconsistencies with the very low or medium EM-RSIII fed is that the concentration of the active ingredients may not be enough to overcome the oxidative stress in the colon. This is not unusual, and commonly used bowel preparations can produce histological abnormalities of the colorectal mucosa. While in the case of high EM-RSIII fed rat samples, the colon showed thick mucosal lining with no sign of infiltration or inflammation related to its unique antioxidant activity compared to native maize flour [12]. The crypts had a nearly normal architecture, and goblet cell component, accompanied by mucin replenishment of high EM-RSIII fed rats, compared with control. In addition to this, the cecum wall length and weight were also examined in control and EM-RSIII fed rats, and results depicted the increase in cecum length and cecum wall weight, especially in the rats fed with medium and high EM-RSIII diet. Similar results were reported by Wu Y *et al*, maximum number of goblet cells, longest crypt depth, and highest level of PYY were found in the distal colon of rats fed higher potato RS diets [32]. In addition to this, similar results were also observed in a study conducted by Sajilata *et al*, on rats RS diet with 25% raw potatoes raised the cecum size and cecum pool of SCFAs, and also elevated the absorption of SCFAs and lowers the plasma triglyceride and cholesterol level [33]. The possible explanation for this is the production of SCFAs due to RS III fermentation by colonic microorganisms, which provides energy to the colonocytes, and the cells grow and renew, affecting the cecum length and weight [34]. In a study conducted on rats, the RS III diet with 25% raw potatoes raised the cecum size and cecum pool of SCFAs, elevated the absorption of SCFAs, and lowered the plasma triglyceride and cholesterol level [35].

The diet-specific microbial shift in fecal and intestinal content was examined during the study along with metabolic or physiological effects. For instance, current study focued to explored the effect of enzymatically prepared prebiotic on a healthy (disease-free) gut microbiome. Whereas, most of the studies focused on prebiotic specific phenotype on any disease, while very few data about prebiotic effect on healthy gut microbiome is available, which is focal point of current study. The increase in the *Lactobacilli* in the intestinal and fecal samples of high fed EM-RSIII rats was observed, attributed to the stabilisation and stimulation of the beneficial microflora in the colon. Amongst the most important genera of colonic bacteria, *Lactobacillus* are accounted as beneficial bacteria and recommended as probiotics. They may improve resistance to disease by lowering the growth of pathogenic and putrefactive bacteria by competing directly for substrate, lowering pH, producing inhibitor molecules and mucosal attachment sites and stimulating the enteric immune system.

Result of the current study investigated that utilization of prebiotic enhanced the rate of butyrate producing microorganism along with improvement in *Lactobacilli*, and *Prevotella* in rat’s fecal microbiome. The prebiotic treatment, overall boosted the microbial diversity by enhancing bacteriods and reduction in firmicutes. Whereas, the enhancement of SCFAs levels in fecal samples designate its role in upgrading gut microbiome dysbiosis in human diseases including obesity, IBDs, colorectal cancer and might serve as potential biotherapy for preventing such diseases.

In the earlier period, a number of studies focused on the beneficial physiological role of *lactobacilli* in the animal colon. According to the investigation of Kleessen *et al*. utilizing unmodified tapioca starch for a feeding period of 180-d observed the variation in cecal aerobic microflora with elevation of *lactobacilli*, while rats feeding with hydroxypropyl starch resulted in complete reduction of *streptococci* [36]. Previous research also showed that taking RS containing food also increase the proportion of microbes like *lactobacillus* and *bifidobacteria* in rats [31, 37]. Whereas, Sáez-Orviz et al has also been confirmed that starch granules get penetrated in the probiotic bacterial cell and the bacteria degraded the starch granules analysed under by electron microscope [38]. These scientific evidence impowered our view that with slight structural modification of starch directed the alteration/variation of intestinal gut microbiome composition. However, even with all these evidence it is very hard to categorized the specific group of microorganism responsible for the physiological changes after administration of modified starch (RS III etc). Because, several gut microorganism utilized the prebiotic (RS III) by complementing other microorganisms in their metabolic activities and by cross-feeding other beneficial non-amylolytic species. Our study suggests that EM-RSIII boosted the occurrence of probiotic bacteria and completely suppressing the growth of *E*. *coli* in high EM-RSIII fed rats.

The *Lactobacilli* was isolated from high EM-RSIII fed rats at somewhat higher frequencies than low and medium EM-RSIII fed rats as compared to control rat’s sample.

## Conclusions

We herein develop a concept to utilise novel prebiotic EM-RSIII, with the potential to prevent the development of uropathogenic strains of *Enterobacteriaceae*. Similarly acute and chronic feeding of selected prebiotic modifies the fecal microbiota and enhance the generation of SCFAs in rat’s gut and feces. This work gives evidence that such EM-RSIII prebiotics could be used as biotherapeutic regimens for numerous human diseases associated with the colon. The current research study could be effective for future research work aimed to explore the influence of prebiotics on the human microbiome, metabolism, and associated diseases.

## Supporting information

S1 Fig Isolation of faecal probiotic bacteria on de Man, Rogosa and Sharpe agar. A: Control fed diet, B: Low EM-RSIII fed diet, C: Medium EM-RSIII fed diet, D: High EM-RSIII fed diet.
(DOCX)

S2 Fig Isolation of faecal probiotic bacteria on modified Columbia agar plates. A: Control fed diet, B: Low EM-RSIII fed diet, C: Medium EM-RSIII fed diet, D: High EM-RSIII fed diet.
(DOCX)

S3 Fig Isolation of cecal probiotic bacteria on modified YCF agar plates. A: Control fed diet, B: Low EM-RSIII fed diet, C: Medium EM-RSIII fed diet, D: High EM-RSIII fed diet.
(DOCX)

S4 Fig Isolation of cecal probiotic bacteria on Rogosa agar plates. A: Control fed diet, B: Low EM-RSIII fed diet, C: Medium EM-RSIII fed diet, D: High EM-RSIII fed diet.
(DOCX)

S5 Fig Isolation of cecal probiotic bacteria on eosin methylene blue agar plates. A: Control fed diet, B: Low EM-RSIII fed diet, C: Medium EM-RSIII fed diet, D: High EM-RSIII fed diet.
(DOCX)

S6 Fig GC-spectra of fecal sample of control fed rat’s sample. (DOCX)

S7 Fig GC-spectra of fecal sample of low EM-RSIII fed rat’s sample. (DOCX)

S8 Fig GC-spectra of fecal sample of medium EM-RSIII fed rat’s sample. (DOCX)

S9 Fig GC-spectra of fecal sample of high EM-RSIII fed rat’s sample. (DOCX)

S1 Table Feed intake of rat’s for 21 days. (PDF)

## References

[1] Slavin J. Fiber and prebiotics: mechanisms and health benefits. Nutrients. 2013 Apr;5(4):1417–35. doi: 10.3390/nu5041417 23609775 PMC3705355

[2] Ley SH, Hamdy O, Mohan V, Hu FB. Prevention and management of type 2 diabetes: dietary components and nutritional strategies. The Lancet. 2014 Jun 7;383(9933):1999–2007. doi: 10.1016/S0140-6736(14)60613-9 24910231 PMC4751088

[3] Cabré E. Clinical Nutrition University: Nutrition in the prevention and management of irritable bowel syndrome, constipation and diverticulosis. e-SPEN, the European e-Journal of Clinical Nutrition and Metabolism. 2011 Apr 1;6(2):e85–95.

[4] Ma Z, Boye JI. Research advances on structural characterization of resistant starch and its structure-physiological function relationship: A review. Critical reviews in food science and nutrition. 2018 May 3;58(7):1059–83. doi: 10.1080/10408398.2016.1230537 27646607

[5] Zi‐Ni T, Rosma A, Napisah H, Karim AA, Liong MT. Characteristics of Metroxylon sagu resistant starch type III as prebiotic substance. Journal of food science. 2015 Apr;80(4):H875–82. doi: 10.1111/1750-3841.12817 25739421

[6] Ludwig DS. The glycemic index: physiological mechanisms relating to obesity, diabetes, and cardiovascular disease. Jama. 2002 May 8;287(18):2414–23. doi: 10.1001/jama.287.18.2414 11988062

[7] Deng Q, Haszard JJ, Conner TS, Rapsey C, Peng M, Venn BJ. Cognitive performance, mood and satiety following ingestion of beverages imparting different glycaemic responses: a randomised double-blind crossover trial. European Journal of Clinical Nutrition. 2021 Apr;75(4):602–10. doi: 10.1038/s41430-020-00749-6 32943769

[8] Öztürk S, Mutlu S. Physicochemical properties, modifications, and applications of resistant starches. InStarches for food application 2019 Jan 1 (pp. 297–332). Academic Press.

[9] Holmbäck U. Insulin Resistance in Pediatric Obesity—Physiological Effects and Possible Diet Treatment. InGlobal Perspectives on Childhood Obesity 2019 Jan 1 (pp. 195–207). Academic Press. doi: 10.1021/acs.est.8b05656

[10] Peng M, Patel P, Nagarajan V, Bernhardt C, Carrion M, Biswas D. Feasible options to control colonization of enteric pathogens with designed synbiotics. In Dietary interventions in gastrointestinal diseases 2019 Jan 1 (pp. 135–149). Academic Press.

[11] Khangwal I, Shukla P. Potential prebiotics and their transmission mechanisms: Recent approaches. Journal of Food and Drug analysis. 2019 Jul 1;27(3):649–56. doi: 10.1016/j.jfda.2019.02.003 31324281 PMC9307030

[12] Khan A, Rahman UU, Siddiqui S, Irfan M, Shah AA, Badshah M et al. Preparation and characterization of resistant starch type III from enzymatically hydrolyzed maize flour. Molecular biology reports. 2019 Aug;46(4):4565–80. doi: 10.1007/s11033-019-04913-5 31243724

[13] Brites CM, Trigo MJ, Carrapiço B, Alviña M, Bessa RJ. Maize and resistant starch enriched breads reduce postprandial glycemic responses in rats. Nutrition Research. 2011 Apr 1;31(4):302–8. doi: 10.1016/j.nutres.2011.02.001 21530804

[14] DeFrance A, Armbruster D, Petty D, Cooper KC, Dasgupta A. Abbott ARCHITECT clinical chemistry and immunoassay systems: digoxin assays are free of interferences from spironolactone, potassium canrenoate, and their common metabolite canrenone. Therapeutic drug monitoring. 2011 Feb 1;33(1):128–31. doi: 10.1097/FTD.0b013e3181fd4c30 21079546

[15] Vento PJ, Swartz ME, Martin LB, Daniels D. Food intake in laboratory rats provided standard and fenbendazole-supplemented diets. Journal of the American Association for Laboratory Animal Science. 2008 Nov 1;47(6):46–50. 19049253 PMC2687130

[16] Bushara YM, Ali AM, Omer MA, Gouda JG, Tet PF, Mohamed MF. Evaluation of Diabetic Rats Behavior after Treatment by Artemisia herba-alba Relative to Insulin. Journal of Diabetes Mellitus. 2017;7(01):1.

[17] Meenu M, Xu B. A critical review on anti-diabetic and anti-obesity effects of dietary resistant starch. Critical reviews in food science and nutrition. 2019 Oct 11;59(18):3019–31. doi: 10.1080/10408398.2018.1481360 29846089

[18] Shen RL, Zhang WL, Dong JL, Ren GX, Chen M. Sorghum resistant starch reduces adiposity in high-fat diet-induced overweight and obese rats via mechanisms involving adipokines and intestinal flora. Food and Agricultural Immunology. 2015 Jan 2;26(1):120–30.

[19] García-Carpintero Fernández-Pacheco S. The gut microbiota pattern predictive for development of Metabolic Syndrome and dietary modulation.

[20] Lockyer S, Nugent AP. Health effects of resistant starch. Nutrition bulletin. 2017 Mar;42(1):10–41.

[21] Olli K, Salli K, Alhoniemi E, Saarinen M, Ibarra A, Vasankari T et al. Postprandial effects of polydextrose on satiety hormone responses and subjective feelings of appetite in obese participants. Nutrition journal. 2015 Dec;14(1):1–2. doi: 10.1186/1475-2891-14-2 25555562 PMC4320494

[22] den BG, Gerding A, van Dijk TH, Ciapaite J, Bleeker A, van Eunen K et al. Protection against the metabolic syndrome by guar gum-derived short-chain fatty acids depends on peroxisome proliferator-activated receptor γ and glucagon-like peptide-1. PLoS One. 2015 Aug 20;10(8):e0136364. doi: 10.1371/journal.pone.0136364 26292284 PMC4546369

[23] Freeland KR, Wolever TM. Acute effects of intravenous and rectal acetate on glucagon-like peptide-1, peptide YY, ghrelin, adiponectin and tumour necrosis factor-α. British Journal of Nutrition. 2010 Feb;103(3):460–6. doi: 10.1017/S0007114509991863 19818198

[24] Coppola S, Avagliano C, Calignano A, Berni Canani R. The protective role of butyrate against obesity and obesity-related diseases. Molecules. 2021 Jan;26(3):682. doi: 10.3390/molecules26030682 33525625 PMC7865491

[25] Romaní-Pérez M, Bullich-Vilarrubias C, López-Almela I, Liébana-García R, Olivares M, Sanz Y. The Microbiota and the Gut–Brain Axis in Controlling Food Intake and Energy Homeostasis. International Journal of Molecular Sciences. 2021 Jan;22(11):5830. doi: 10.3390/ijms22115830 34072450 PMC8198395

[26] Wang Y, Alkhalidy H, Liu D. The Emerging Role of Polyphenols in the Management of Type 2 Diabetes. Molecules. 2021 Jan;26(3):703. doi: 10.3390/molecules26030703 33572808 PMC7866283

[27] Behall KM, Scholfield DJ. Food amylose content affects postprandial glucose and insulin responses. Cereal Chemistry. 2005 Nov;82(6):654–9.

[28] Aziz AA, Kenney LS, Goulet B, Abdel-Aal ES. Dietary starch type affects body weight and glycemic control in freely fed but not energy-restricted obese rats. The Journal of nutrition. 2009 Oct 1;139(10):1881–9. doi: 10.3945/jn.109.110650 19692526

[29] McNabney SM, Henagan TM. Short chain fatty acids in the colon and peripheral tissues: a focus on butyrate, colon cancer, obesity and insulin resistance. Nutrients. 2017 Dec;9(12):1348. doi: 10.3390/nu9121348 29231905 PMC5748798

[30] Han KH, Fukushima M, Kato T, Kojima M, Ohba K, Shimada KI et al. Enzyme-resistant fractions of beans lowered serum cholesterol and increased sterol excretions and hepatic mRNA levels in rats. Lipids. 2003 Sep;38(9):919–24. doi: 10.1007/s11745-003-1145-2 14584599

[31] Wu Y, Hu H, Dai X, Che H, Zhang H. Effects of dietary intake of potatoes on body weight gain, satiety-related hormones, and gut microbiota in healthy rats. RSC advances. 2019;9(57):33290–301. 35529109 10.1039/c9ra04867g PMC9073283

[32] Sajilata MG, Singhal RS, Kulkarni PR. Resistant starch–a review. Comprehensive reviews in food science and food safety. 2006 Jan;5(1):1–7. doi: 10.1111/j.1541-4337.2006.tb00076.x 33412740

[33] Alrashidi N.A., Zafar T.A. and Khan I., 2021. High‐Amylose Cornstarch Variably Affects Food Intake and Body Composition of Rats When Substituted to Standard versus a Moderately High‐Fat High‐Sugar Diet. *Starch‐Stärke*, 73(3–4), p.2000036.

[34] Martinez-Flores HE, Chang YK, Martinez-Bustos F, Sgarbieri V. Effect of high fiber products on blood lipids and lipoproteins in hamsters. Nutrition research. 2004 Jan 1;24(1):85–93.

[35] Lopez HW, Levrat-Verny MA, Coudray C, Besson C, Krespine V, Messager A et al. Class 2 resistant starches lower plasma and liver lipids and improve mineral retention in rats. The Journal of nutrition. 2001 Apr 1;131(4):1283–9. doi: 10.1093/jn/131.4.1283 11285339

[36] Kleessen B, Stoof G, Proll J, Schmiedl D, Noack J, Blaut M. Feeding resistant starch affects fecal and cecal microflora and short-chain fatty acids in rats. Journal of animal science. 1997 Sep 1;75(9):2453–62. doi: 10.2527/1997.7592453x 9303464

[37] Wang Z., Lin Y., Liu L. et al. 2022. Effect of Lotus Seed Resistant Starch on Lactic Acid Conversion to Butyric Acid Fermented by Rat Fecal Microbiota. *Journal of Agricultural and Food Chemistry*. doi: 10.1021/acs.jafc.1c06000 34989559

[38] Sáez-Orviz S., Marcet I., Rendueles M. et al. 2021. Bioactive packaging based on delipidated egg yolk protein edible films with lactobionic acid and Lactobacillus plantarum CECT 9567: Characterization and use as coating in a food model. *Food Hydrocolloids*, 119, p.106849.

