# Supplementary figures and images for: Prebiotic potential of enzymatically prepared resistant starch in reshaping gut microbiota and their respond to body physiology
Source: PLoS One. 2022 May 16;17(5):e0267318. doi: 10.1371/journal.pone.0267318 (PMC9109903; doi:10.1371/journal.pone.0267318)

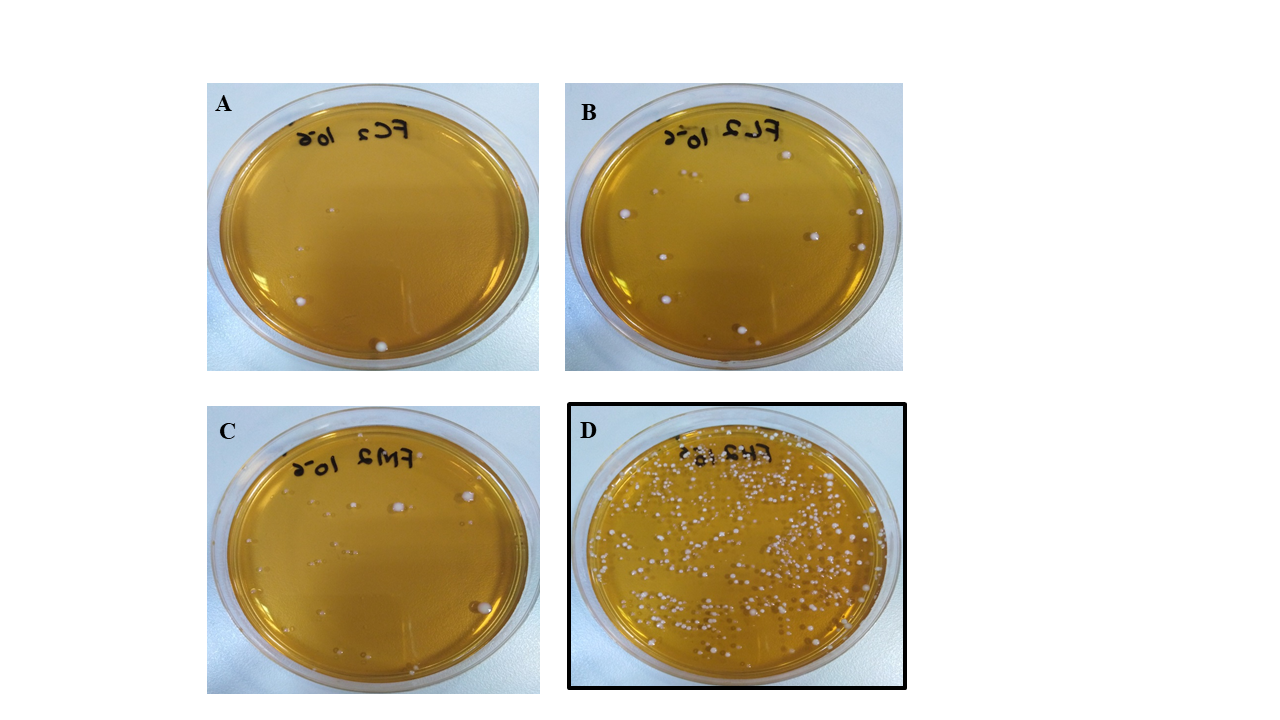


S1_Fig

Supplement: S1 Fig — A: Control fed diet, B: Low EM-RSIII fed diet, C: Medium EM-RSIII fed diet, D: High EM-RSIII fed diet. (DOCX) [file pone.0267318.s001.docx]

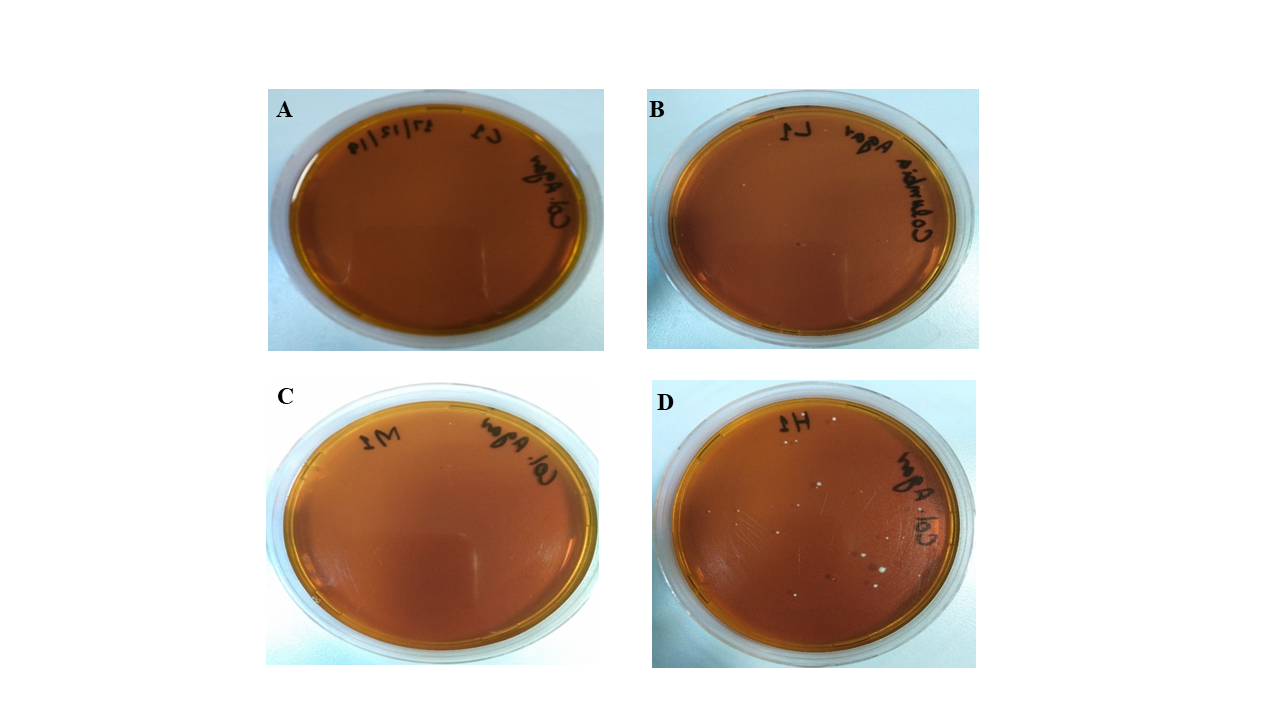


S2_Fig

Supplement: S2 Fig — A: Control fed diet, B: Low EM-RSIII fed diet, C: Medium EM-RSIII fed diet, D: High EM-RSIII fed diet. (DOCX) [file pone.0267318.s002.docx]

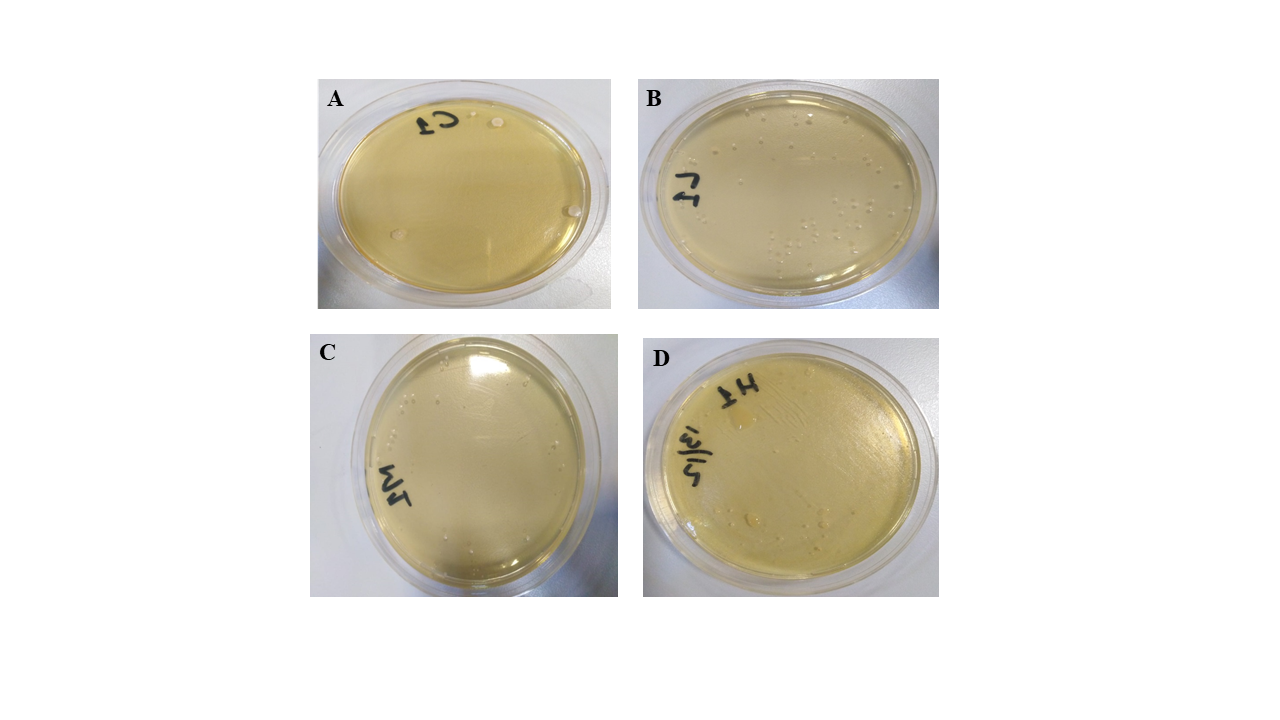


S3_Fig

Supplement: S3 Fig — A: Control fed diet, B: Low EM-RSIII fed diet, C: Medium EM-RSIII fed diet, D: High EM-RSIII fed diet. (DOCX) [file pone.0267318.s003.docx]

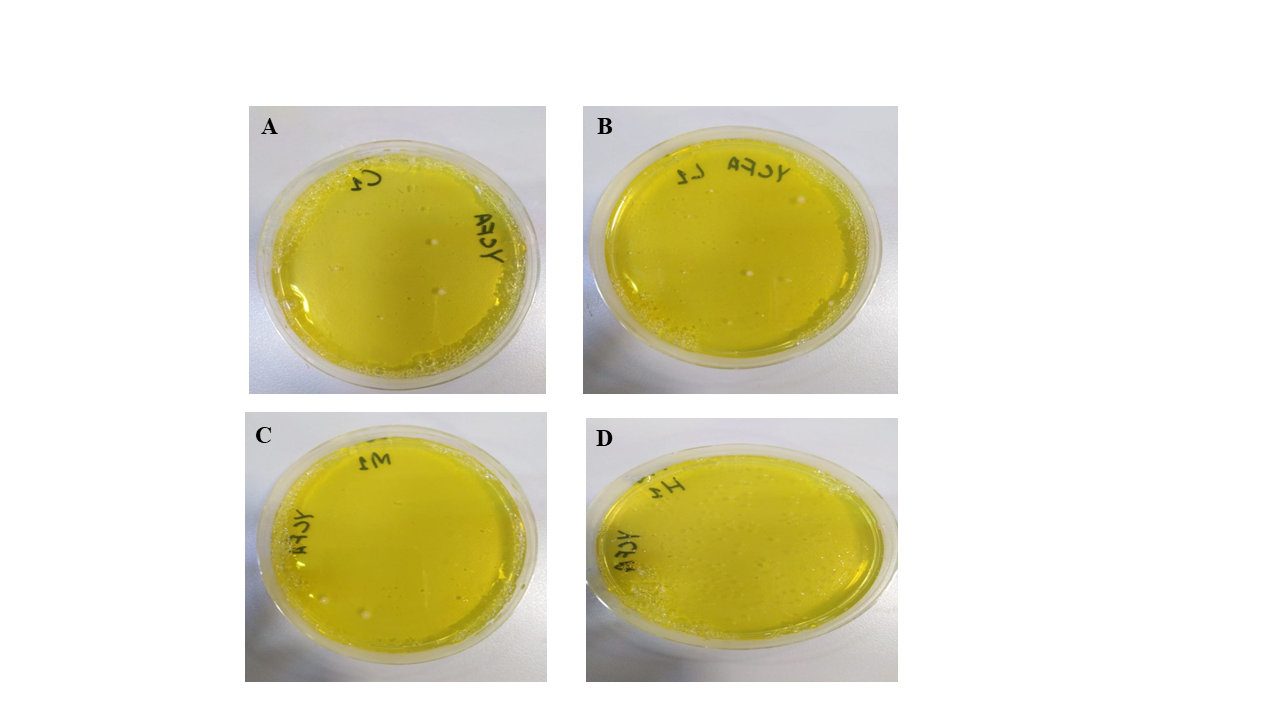


S4_Fig

Supplement: S4 Fig — A: Control fed diet, B: Low EM-RSIII fed diet, C: Medium EM-RSIII fed diet, D: High EM-RSIII fed diet. (DOCX) [file pone.0267318.s004.docx]

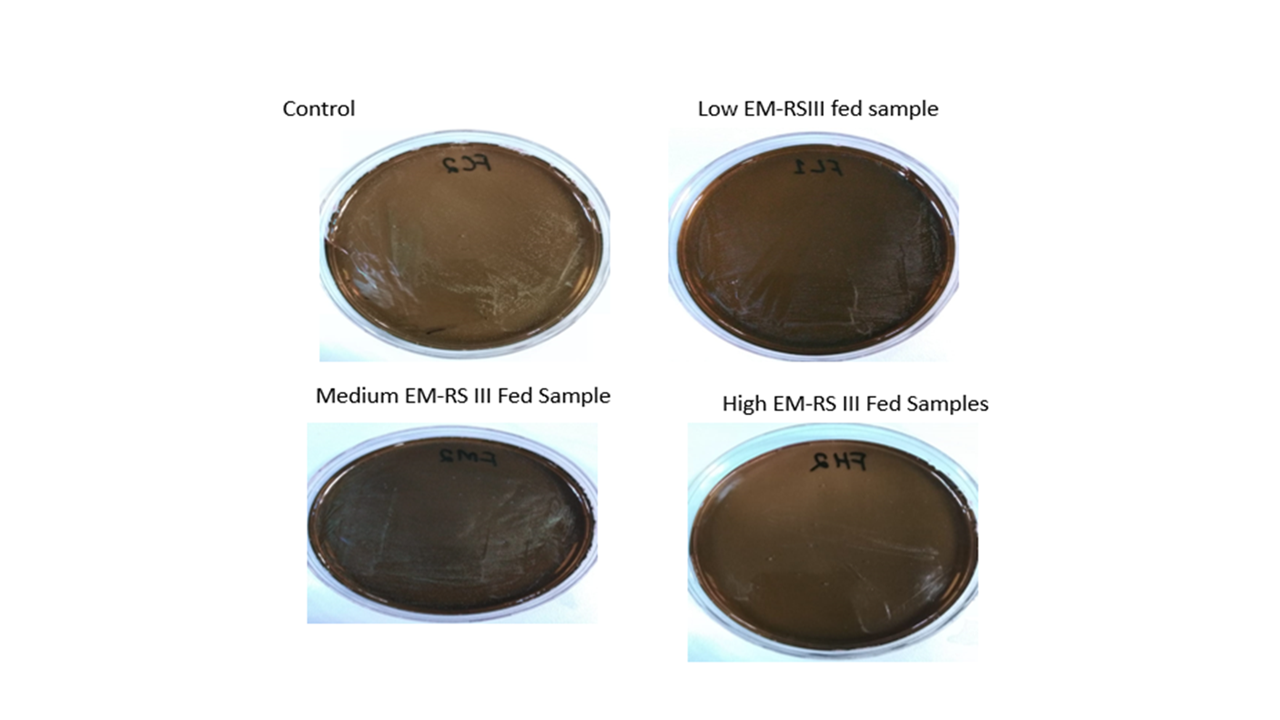


S5_Fig

Supplement: S5 Fig — A: Control fed diet, B: Low EM-RSIII fed diet, C: Medium EM-RSIII fed diet, D: High EM-RSIII fed diet. (DOCX) [file pone.0267318.s005.docx]

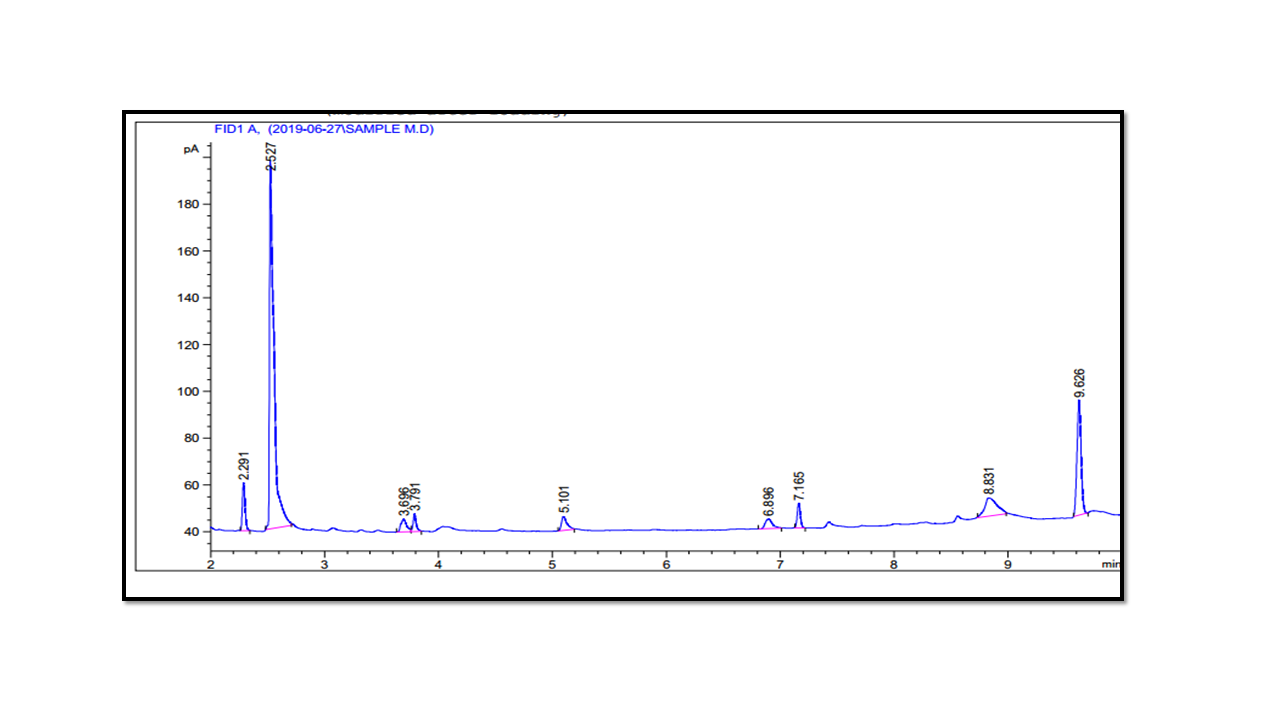


S6_Fig

Supplement: S6 Fig — (DOCX) [file pone.0267318.s006.docx]

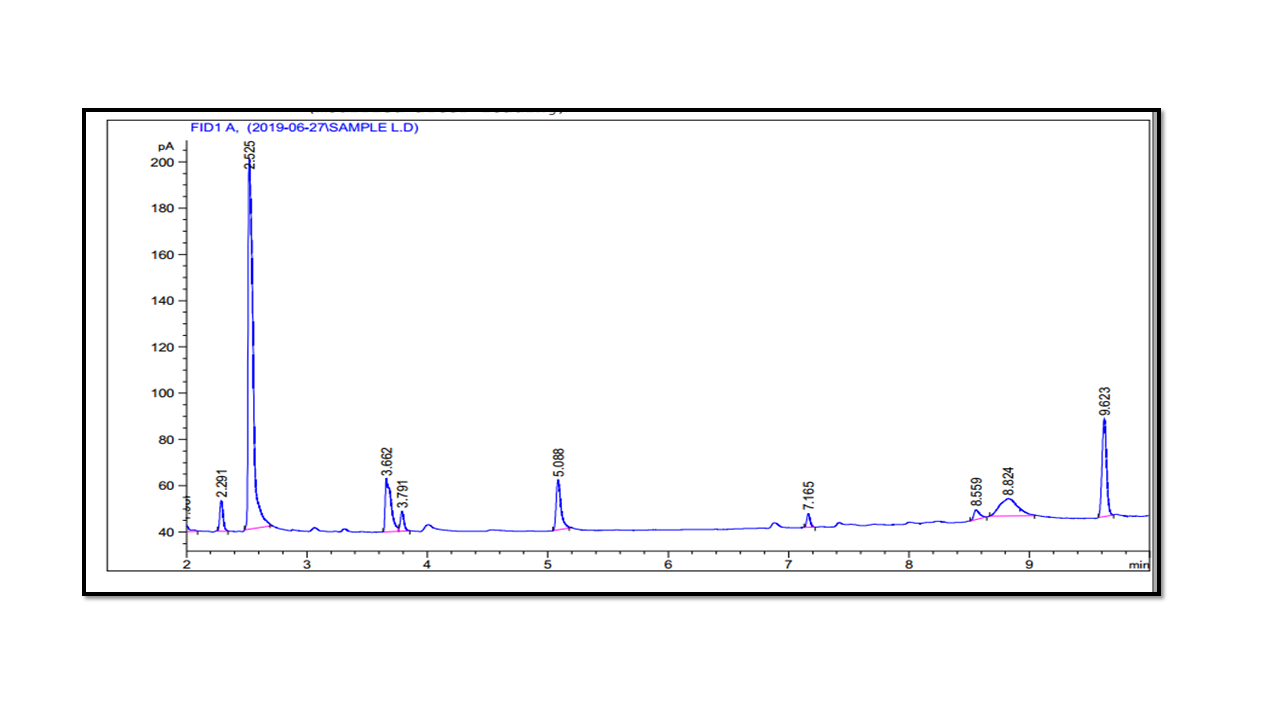


S7-Fig

Supplement: S7 Fig — (DOCX) [file pone.0267318.s007.docx]

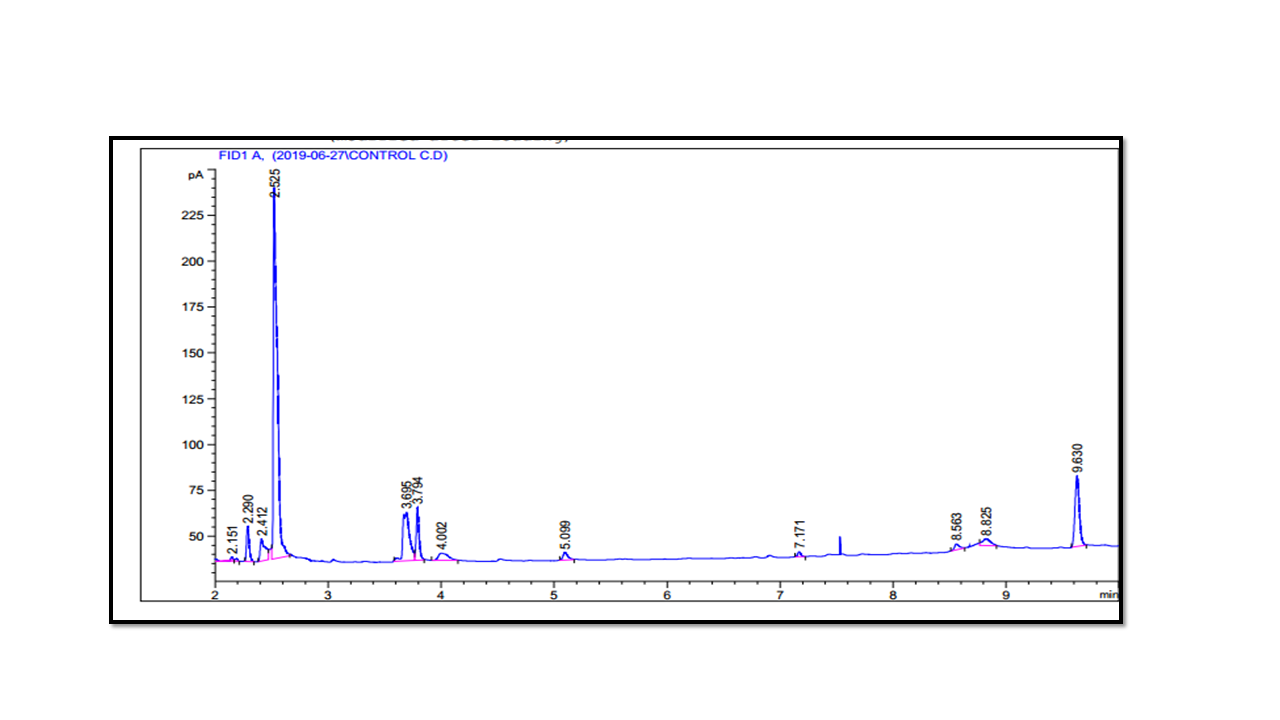


S8_Fig

Supplement: S8 Fig — (DOCX) [file pone.0267318.s008.docx]

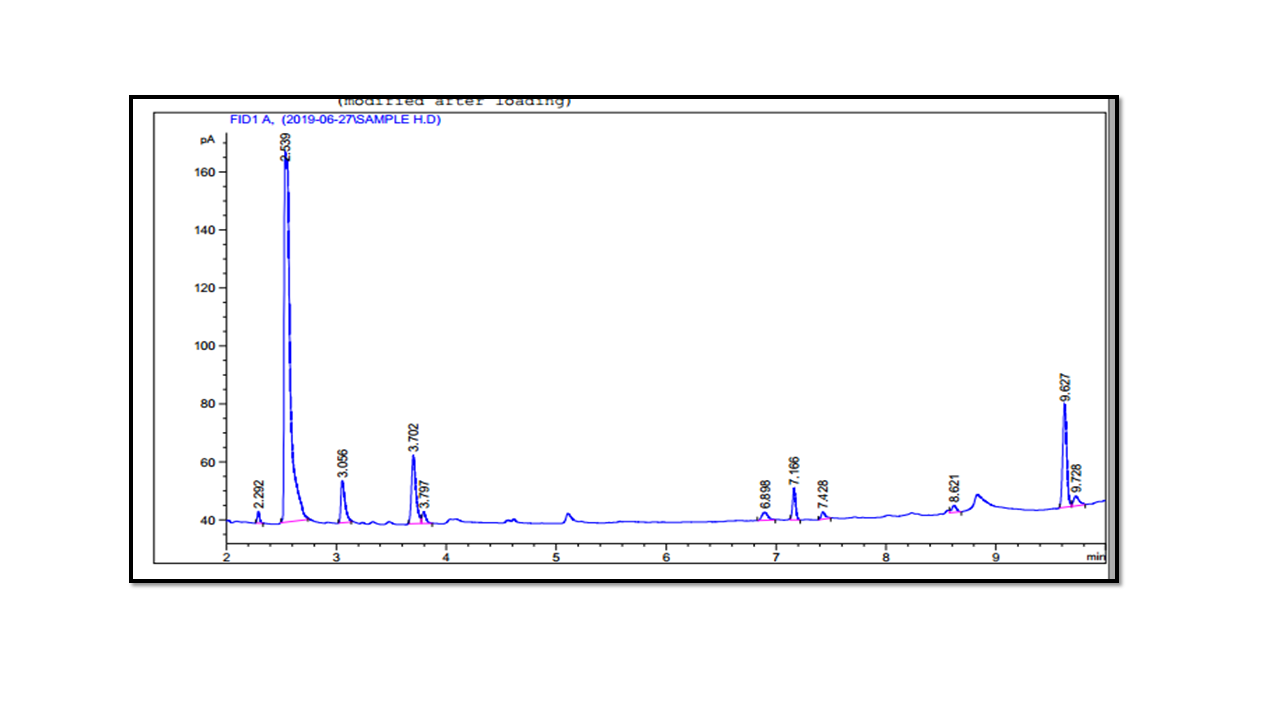


S9_Fig

Supplement: S9 Fig — (DOCX) [file pone.0267318.s009.docx]
