# Supplementary material for: Prebiotic potential of enzymatically prepared resistant starch in reshaping gut microbiota and their respond to body physiology
Source: PLoS One. 2022 May 16;17(5):e0267318. doi: 10.1371/journal.pone.0267318 (PMC9109903; doi:10.1371/journal.pone.0267318)
Supplement: S1 Table — (PDF) [file pone.0267318.s010.pdf]

S1 Table: Feed Intake of rat’s for 21 days

Days	Control Feed intake g/day	Low EM-RSIII Feed intake g/day	Medium EM-RSIII Feed intake g/day	High EM-RSIII Feed intake g/day
**1**	99.83±0.29a	99.57±0.12a	99.17±0.58a	90.33±0.29b
**2**	99.83±0.29a	99.17±0.29b	99.17±0.58ab	90.17±0.29c
**3**	100.39±0.35a	99.17±0.29b	98.83±0.29ab	88.83±0.29c
**4**	99.67±0.58a	99.17±0.29b	98.17±0.29c	88.33±0.58d
**5**	99.67±0.58a	98.60±0.17b	97.33±0.29c	87.17±0.29d
**6**	99.67±0.58a	98.33±0.29b	96.67±0.29c	86.33±0.29d
**7**	100.00±0.39a	98.10±0.17b	95.83±0.29c	86.17±0.29d
**8**	99.83±0.29a	98.17±0.29b	95.67±0.29c	85.33±0.29d
**9**	99.67±0.29a	97.57±0.12b	94.83±0.29c	85.00±0.00d
**10**	100.00±0.00a	97.33±0.29b	94.67±0.29c	84.33±0.29d
**11**	99.67±0.29a	97.17±0.29b	94.17±0.29c	83.83±0.29d
**12**	99.83±0.29a	96.33±0.29b	94.17±0.29c	83.67±0.29d
**13**	99.83±0.29a	96.67±0.29b	93.33±0.29c	83.00±0.00d
**14**	100.00±0.41a	95.40±0.17b	92.83±0.29c	82.67±0.29d
**15**	99.67±0.58a	95.60±0.17b	92.67±0.29c	82.17±0.29d
**16**	99.67±0.58a	95.40±0.17b	91.83±0.29c	81.37±0.23d
**17**	99.67±0.58a	95.17±0.14b	91.33±0.29c	81.10±0.17d
**18**	100.00±0.51a	95.17±0.14b	91.17±0.29c	80.63±0.23d
**19**	99.83±0.29a	95.03±0.06b	90.10±0.17c	80.23±0.40d
**20**	99.67±0.29a	95.13±0.23b	89.83±0.29c	80.10±0.17d
**21**	100.00±0.38a	94.87±0.23b	90.10±0.17c	80.17±0.29d

Mean (n=5) with the same letter in a column within same water regime are statistically similar at $p < 0.05$ according to Duncan’s multiple range test. Low RS = 2g/100g, Medium RS= 4/100g, High RS= 8g/100g.
